# Altered Reward Circuitry in the Norepinephrine Transporter Knockout Mouse

**DOI:** 10.1371/journal.pone.0057597

**Published:** 2013-03-04

**Authors:** Joseph J. Gallagher, Xiaowei Zhang, F. Scott Hall, George R. Uhl, Elaine L. Bearer, Russell E. Jacobs

**Affiliations:** 1 Biological Imaging Center, Beckman Institute, California Institute of Technology, Pasadena, California, United States of America; 2 Department of Pathology, University of New Mexico Health Sciences Center, Albuquerque, New Mexico, United States of America; 3 Molecular Neurobiology Branch, National Institute on Drug Abuse, Intramural Research Program, Baltimore, Maryland, United States of America; Kent State University, United States of America

## Abstract

Synaptic levels of the monoamine neurotransmitters dopamine, serotonin, and norepinephrine are modulated by their respective plasma membrane transporters, albeit with a few exceptions. Monoamine transporters remove monoamines from the synaptic cleft and thus influence the degree and duration of signaling. Abnormal concentrations of these neuronal transmitters are implicated in a number of neurological and psychiatric disorders, including addiction, depression, and attention deficit/hyperactivity disorder. This work concentrates on the norepinephrine transporter (NET), using a battery of *in vivo* magnetic resonance imaging techniques and histological correlates to probe the effects of genetic deletion of the norepinephrine transporter on brain metabolism, anatomy and functional connectivity. MRS recorded in the striatum of NET knockout mice indicated a lower concentration of NAA that correlates with histological observations of subtle dysmorphisms in the striatum and internal capsule. As with DAT and SERT knockout mice, we detected minimal structural alterations in NET knockout mice by tensor-based morphometric analysis. In contrast, longitudinal imaging after stereotaxic prefrontal cortical injection of manganese, an established neuronal circuitry tracer, revealed that the reward circuit in the NET knockout mouse is biased toward anterior portions of the brain. This is similar to previous results observed for the dopamine transporter (DAT) knockout mouse, but dissimilar from work with serotonin transporter (SERT) knockout mice where Mn^2+^ tracings extended to more posterior structures than in wildtype animals. These observations correlate with behavioral studies indicating that SERT knockout mice display anxiety-like phenotypes, while NET knockouts and to a lesser extent DAT knockout mice display antidepressant-like phenotypic features. Thus, the mainly anterior activity detected with manganese-enhanced MRI in the DAT and NET knockout mice is likely indicative of more robust connectivity in the frontal portion of the reward circuit of the DAT and NET knockout mice compared to the SERT knockout mice.

## Introduction

Norepinephrine (NE) is a monoamine neurotransmitter implicated in various behavioral and psychological functions including learning and memory, anxiety, arousal, and mood; as well as disorders related to these processes (*e.g.* addiction, depression, attention deficit/hyperactivity disorder) [1]–[5]. NE innervation for much of the brain comes from cell bodies of the locus coeruleus (LC). These neurons have diffuse projections to many brain regions with particularly dense innervation in limbic regions, as well as the frontal cortex, and other monoaminergic nuclei (*i.e.* serotonergic raphe nuclei and dopaminergic ventral tegmental area).

The norepinephrine transporter (NET, SLC6A2) is responsible for norepinephrine reuptake by the presynaptic terminal. Thus, it removes NE from the synaptic cleft and terminates noradrenergic neurotransmission, while re-charging presynaptic cells for future transmission. NET is a direct target of both antidepressants and psychostimulants [6], [7]. Additionally, NET mediates dopamine uptake in the prefrontal cortex [8]–[10]. Recent work in animal models has suggested that the mechanism of drugs that treat ADHD may include inhibition of fronto-cortical NET [11], [12].

NE and NET, along with two other monoamines and their transporters (DAT: dopamine transporter, SERT: serotonin transporter) form a complex interacting system that influences a broad range of affective states. Mouse knockouts for NET, DAT, and SERT have been used to study the pharmacological, behavioral, and anatomical consequences of disruption of these monoamine transporters [11], [13]–[19]. Single and multiple knockouts have been especially useful in investigations parsing the molecular actions and behavioral consequences of drugs of abuse [17], [20]–[22]. There is now specific information about several aspects of these rodent model systems at physiological (*e.g*. up/down regulation of monoamine receptors in response to uptake inhibition, altered concentrations of monoamine metabolites and related molecules) and behavioral levels (*e.g*. conditioned place preference, locomotor response, drug induced response) [19], [23]–[27]. These genetically altered mice provide rich model systems for assessing differential effects of cocaine on each transporter [15], [19], [28]–[33] and other biological ramifications of the loss of activity of one or more monoamine transporters [14], [17], [20], [22], [34]–[39].

In previous work we examined consequences of DAT and SERT KO on the functional connectivity of prefrontal cortex neuronal circuitry, brain morphology, and metabolite levels [40], [41]. While there is some discussion about the exact definition of ‘reward circuits’ [42]–[45], one generally accepted part of this idea includes a network linking brainstem and meso-cortical-limbic structures. In this work we concentrate on the prefrontal-ventral striatopallidal circuit implicated in disorders involving the reward pathway and executive functions. Localized injection of manganese, an MR contrast agent well-established as a circuitry tracer [46]–[48], into the prefrontal cortex (PFC) followed by time-lapse whole brain MR imaging enabled quantification of the functional connectivity of the circuitry. We have previously reported more robust connectivity between the PFC and posterior structures in SERT KO mice than in wildtype mice. In DAT KO mice the same procedure revealed preservation of cortico-striatal-thalamic connectivity, but diminished robustness in reward circuitry distal to the thalamus. Structural and metabolic MR analysis revealed no significant differences between these knockouts and wildtype controls. Hence we concluded that lifelong transporter deficits primarily affect the degree of activity in these circuits without major large scale anatomical or metabolic disturbances.

Here we extend this investigation to the NET KO mouse using the same experimental approach as for the other two monoamine transporter knockout strains, which completes our examination of the effects of gene knockout of the three major molecular targets of cocaine. Magnetic resonance spectroscopy (MRS) of the striatum was used to measure the concentration of several small molecule metabolites present at millimolar or greater concentrations that have been associated with disease states [49]–[57]. Diffusion tensor imaging (DTI) was employed to investigate differences in brain structure between NET KO and WT littermate mice using tensor based morphometry to identify local structural differences between the KO and WT mice [58], [59]. We then assessed functional connectivity of neuronal circuitry emanating from the prefrontal cortex using manganese enhanced MRI (MEMRI) after stereotaxic injection of Mn^2+^ into the PFC in NET KO and wildtype (WT) mice.

Co-registration of *in vivo* time-lapse 3D MRI scans and pair-wise group analysis generated statistical parametric maps (SPM) providing unbiased, voxel-wise comparisons of Mn^2+^ signal throughout the entire brain. SPM analysis of Mn^2+^ accumulation distal to the injection site, as a function of time, enabled identification of functional connectivity in multi-synaptic circuits that emanate from the PFC injection site.

This study reports the effects of life-long deletion of NET on metabolite levels, brain structure and neuronal connectivity in the meso-cortical-limbic reward circuitry. These findings are discussed in the context of similar work in previous studies using SERT and DAT KO mice, providing a unique framework to consider the roles of these neurotransmitters in brain function and dysfunction.

## Results

### Reduced NAA concentration in NET KO mice is associated with histological aberrations in the striatal-global pallidus interface

**Figure 1:**
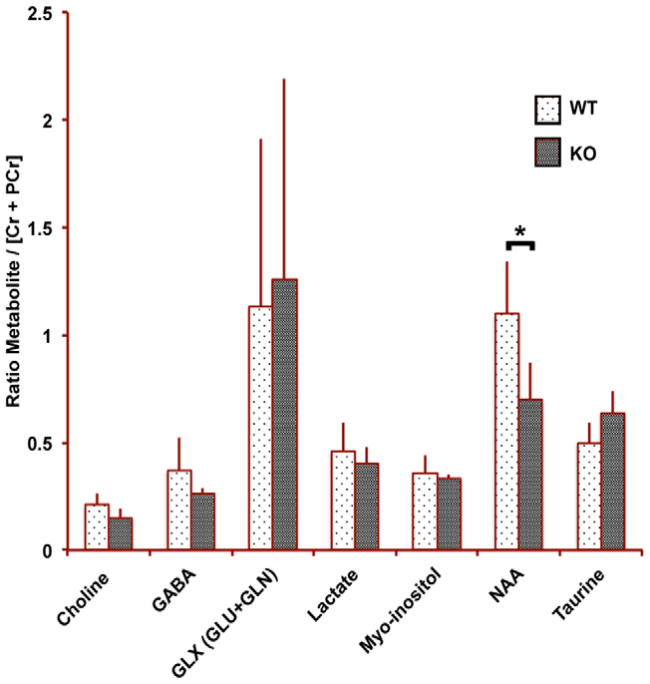
NAA is significantly reduced in NET KO mice. Summary of results from MRS showing metabolite concentrations in the striatum relative to creatine plus phosphocreatine are shown for both cohorts of mice. N-acetyl aspartate (NAA) is significantly different (P<0.05; Student's t-Test for independent means; n = 11–12) between genotypes, with NAA for NET KO mice being 36% less than the wildtype mice.

We used magnetic resonance spectroscopy (MRS) to measure metabolite concentrations in the striatum. Concentrations of metabolites were determined relative to creatine plus phosphocreatine, which is assumed not to vary among mice or between animals of these two genotypes [60]–[62]. Relative concentrations for each metabolite are shown in Figure 1. Student's t-test indicated that of all the metabolite levels only N-acetyl aspartate (NAA) was statistically different in the NET KO versus WT littermate comparison (p<0.05; Students t-Test for Independent means). NAA in the NET KOs was 36% lower than in WT littermates. All other comparisons had p>0.10.

**Figure 2:**
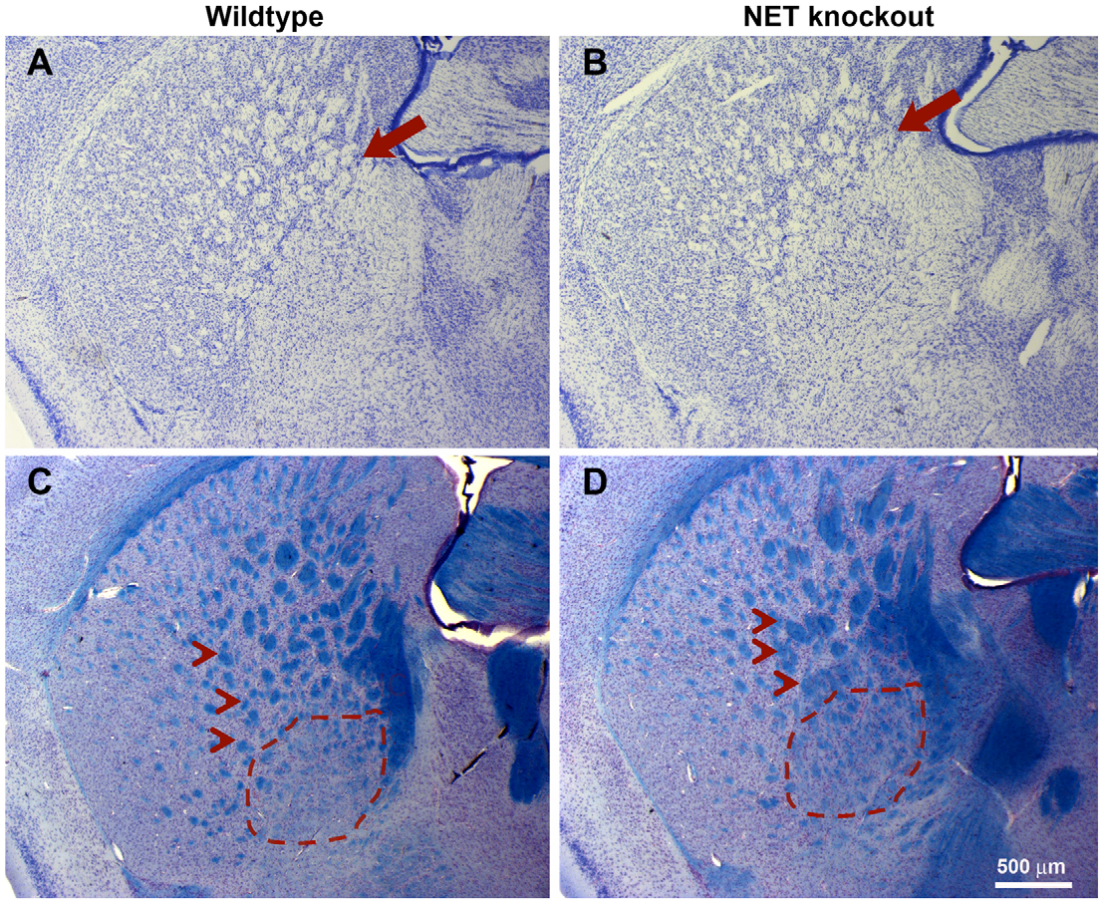
NET KO mice display a disorganized Striatal-GP interface. Representative sections from a wildtype littermate (A and C) and a NET KO mouse that were embedded in register in the same block, sectioned and stained for thionine-Nissl (nuclei; A and B) and Solochrome (C and D). Three animals from each genotype were included in the same block, sectioned in register and stained in the same cup to ensure consistency and accuracy. This co-embedding, co-sectioning procedure yields the highest degree of precision correlations currently available for co-registration of histologic sections, which is of necessity less precise than MR dataset computational alignment. Despite this caveat, a distinct difference in axonal organization and bundling is observed in all 6 brains examined. In (A–B) Arrows indicate the striatum/globus pallidus interface. Note the size and regularity of the axonal swirls in (A) and the lack of regularity in (B). In (C–D) the blue-stained axonal bundles in the striatum of wildtype (arrow heads) are of uniform size and shape while in the KO (D) they are irregularly sized bundles. Also note the presence of scattered bundles in the globus pallidus (GP, outlined with dashed lines) in KO mouse (D) that are not seen in WT littermate (C). The lateral boundary of the internal capsule (IC) is less distinct in the KO mouse as well. While in these representative images some of these differences between WT and KO mice are accentuated by a slightly different angle of the histologic section, such alterations in KO mice were obvious in all three specimens regardless of slight differences in the sectioning plane.

Histological evaluation through the same region in which metabolite concentrations were measured revealed distinct differences in neuronal organization at the interface between the striatum and globus pallidus (Figure 2, red arrows). NET KO mice demonstrated a poorly demarcated interface between the striatum and globus pallidus, with axonal bundles displaying greater variation in size and shape than in wildtype littermate control mice (Figure 2, red arrows). In addition, the lateral boundaries between the fiber bundles of the internal capsule and the striatum displayed reduced regularity, showing an uneven distribution of fibers passing through the striatum and GP. We examined sections through the GP in three mice of each genotype perfused after conclusion of the MRI studies that were immunohisotchemically stained with anti-choline-acetyl transferase (ChAT) to identify cholinergic neurons. Aggregates of ChAT-positive neurons typically line the interface between GP and other brain areas. In the NET−/− mice, cholinergic neurons appeared to be somewhat disorganized at the periphery of the GP and some were even found within the GP (see Figure S1). Such differences in neuronal types and axonal bundling could contribute to the NAA differences between wildtype and knockout animals that we have found in spectroscopic studies in this region.

### Tensor-based morphometry reveals minimal anatomical differences between NET KO and wildtype littermate mice

**Figure 3:**
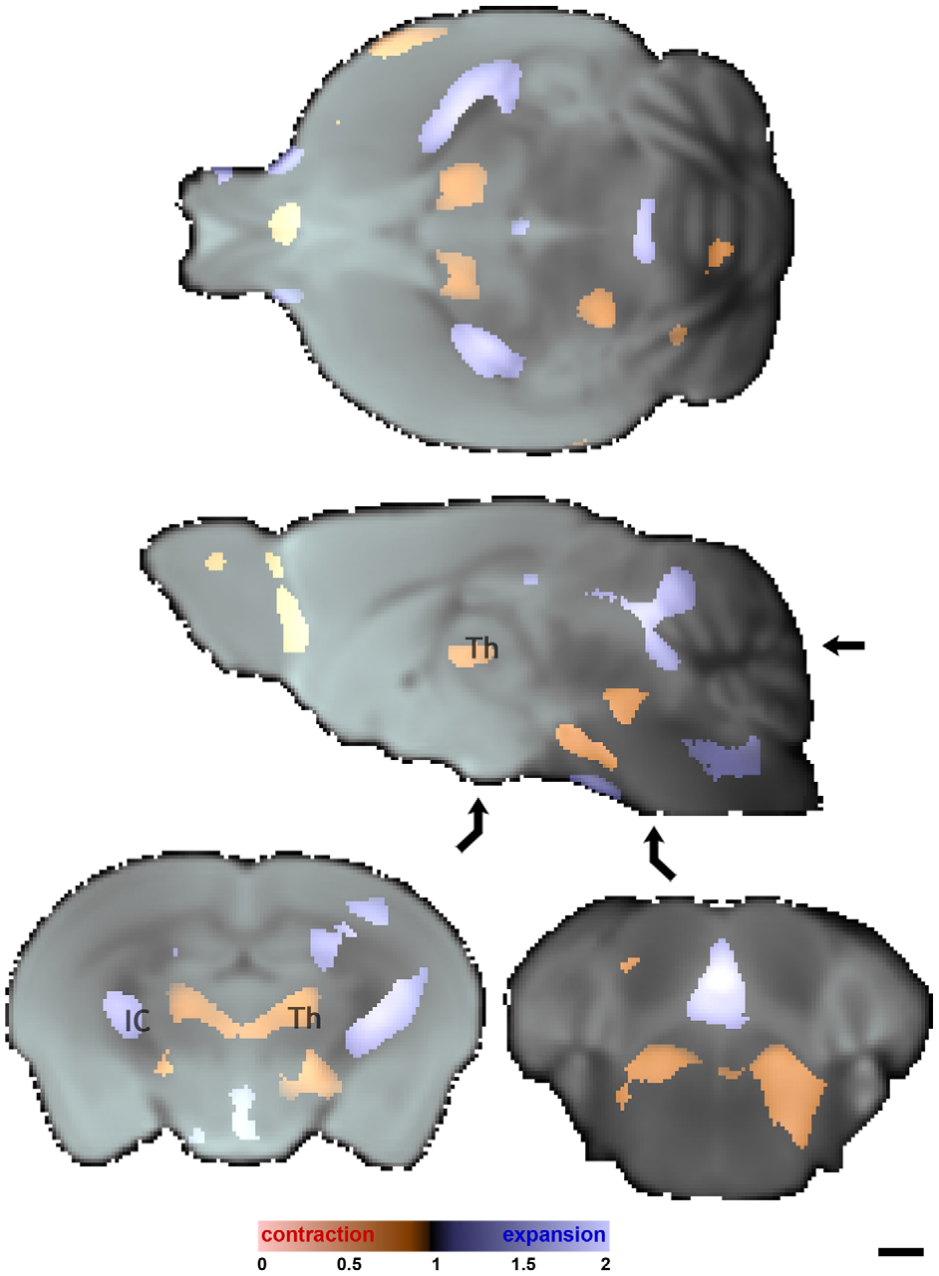
Tensor based morphometry indicates small local volume changes between NET KO mice and wildtype littermates. FDR corrected at p<0.005 statistical parametric maps identifying local fractional volume changes necessary to transform NET KO to wildtype; red denotes regions where NET KO is contracted relative to wildtype and blue denotes regions where NET KO is expanded relative to wildtype. Color bar to the left indicates local fractional volume changes (e.g. 1 implies no change, 0.5 indicates 50% reduction, 2 indicates factor of 2 expansion). Arrows on the sagittal slice indicate locations of horizontal and axial slices. The thalmus (Th) and internal capsule (IC) are indicated. Other specific anatomical regions can be identified using Hoff et al. [109], the Allen Brain Atlas [110] (http://www.brain-map.org/welcome.do), or The Mouse Brain Library (http://www.mbl.org/atlas/atlas.php). Scale bar  = 1 mm.

To gauge the extent of anatomical morphometric alterations associated with NET deletion, we used tensor based morphometry (TBM) and high contrast data sets [58], [63]–[69] (Figure 3). Input to the TBM pipeline was provided by the isotropic diffusion weighted (iDWI) data sets from NET KO and WT littermate mice obtained on fixed specimens following completion of *in vivo* MEMRI experiments. Total brain volumes for the NET KO and WT mice were not significantly different: 452±18 mm^3^ and 465±15 mm^3^, respectively. TBM provides deformation tensor indices that reflect the extent of expansion and contraction that would be required to make a WT brain similar to an NET KO brain. Since this information is provided at every voxel, it allows inspection of areas that are bigger or smaller in the NET KO brains. Relatively few voxels displayed deformation tensor indices that differed between NET KO and WT mice. At a FDR corrected p<0.005 level of significance, approximately 4% of the voxels in NET KO mice were (nominally) significantly expanded, with mean expansion factor of 1.25, and 2% were contracted relative to wildtype mice, with a mean contraction factor of 0.82. Expansion (red) and contraction (blue) of the NET KO relative to wildtype littermates, respectively, displayed coarse left-right symmetry (Figure 3 and Video S2). Expansions occurred in the more exterior parts of the brain and contractions in more interior regions. The midline olfactory bulb was marginally contracted, while piriform and anterior olfactory cortices were expanded beginning approximately 2 mm anterior to bregma. Beginning 1 mm posterior to bregma, the internal capsule was expanded through to the cerebral peduncle, while adjacent ventromedial and ventrolateral thalami were contracted. Further posterior (∼4 mm posterior to bregma) the midline dorsal cerebellar lobules, aqueduct and periaqueductal gray, and brainstem were expanded while the pontine reticular nucleus was contracted. Anterior regions of the interior cerebellum near the midline were contracted in the NET KO compared to wildtype mice; there is apparently compensating expansion of the cerebellum in bordering regions nearer the brain surface. Cortical regions are essentially identical in volumes in mice of the two genotypes studied.

### Functional circuitry from medial forebrain to posterior structures is altered in NET KO mice

#### 

##### **Consistent placement of the injection**

**Figure 4:**
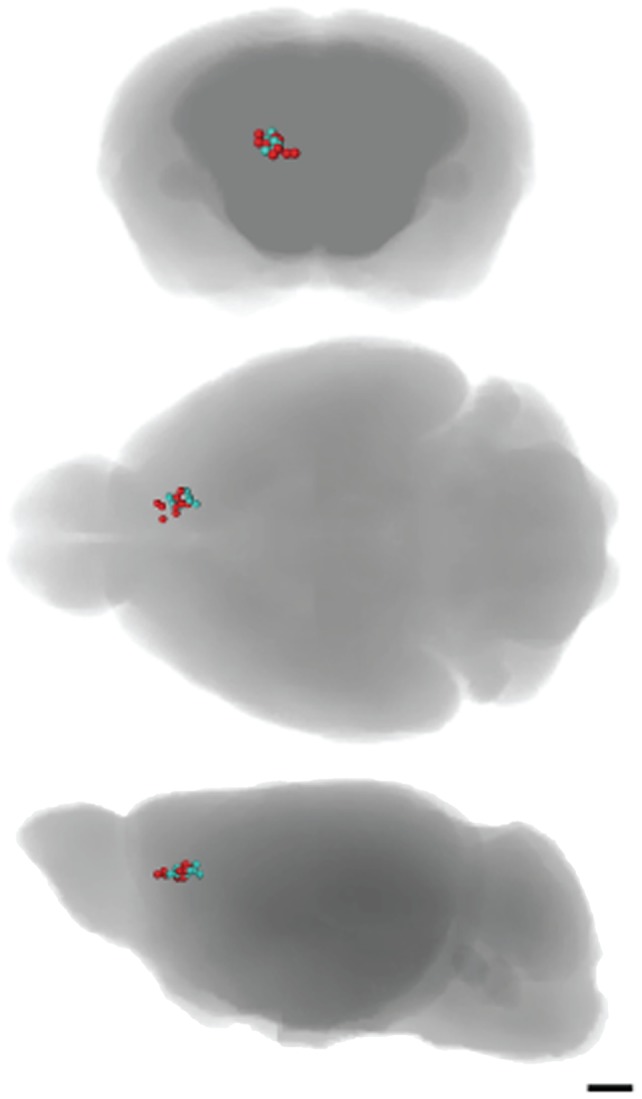
Injection site locations are similar in wildtype and NET KO mice. Injection site locations, represented as a sphere for each animal of the 23 mice used in the statistical analysis of prefrontal cortex injections, confirms accurate placement for both groups (blue: wildtype littermates, red: NET KO). Injection site locations have been overlaid onto axial, transverse and sagittal semi-transparent rendered images of the MRI template (Scale bar  = 1 mm).

**Figure 5:**
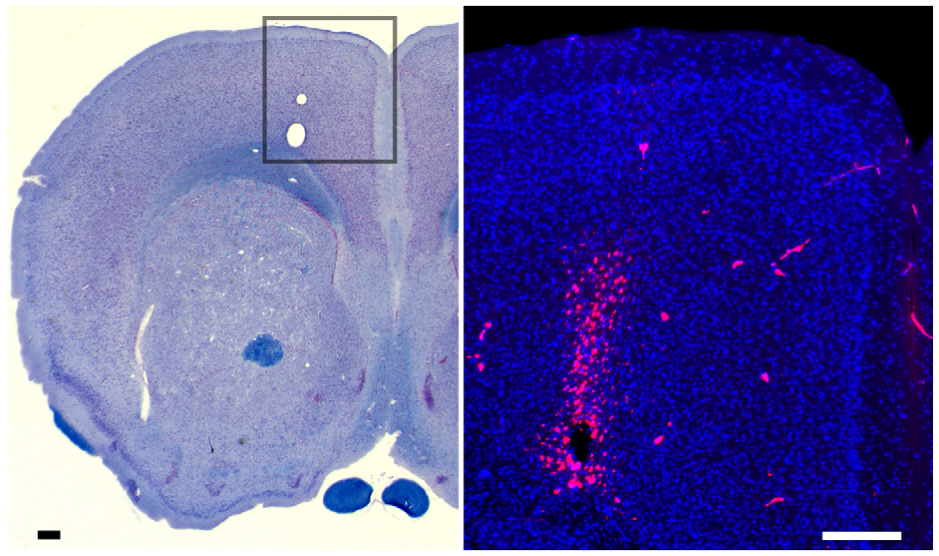
Histology of the injection site showing precise location and minimal damage. ***Left panel:*** A representative example of one brain section through the forebrain stained with Solochrome for both nuclei and fiber tracks and imaged at low magnification shows the injection site in the right frontal cortex (boxed region). Although the co-injected oil droplet identifying the injection site, leaves a small displacement, there is no bleeding, gliosis, or other indication of injury. Scale bar  = 200 μm. ***Right panel:*** Fluorescent micrograph of an adjacent section of the same brain. The injection site is detected by fluorescent signal from the co-injected rhodamine-dextran tracer (red). Note that the tracer is confined to the region of the oil droplet and a few microns above it. Some auto-fluorescence of red blood cells within parenchymal capillaries is scattered in the background. Anatomical features revealed by a counter-stain for cellular nuclei with DAPI (blue) confirm that the location is within the forebrain. Scale bar  = 20 μm.

The center of each injection site fell within a 0.6 mm radius for all 23 animals, as measured in aligned MR images scanned at 30 min post-injection (Figure 4). The average injection site (± standard deviation) was: x (lateral to the midline) +0.85±0.18 mm; y (anterior-posterior with reference to Bregma) +1.43±0.32 mm; z (dorsal-ventral from the brain surface) −1.57±0.24 mm. Histological examination of the injection site revealed no bleeding or gliosis due to the injection procedure, although the co-injected oil droplet was apparent (Figure 5). The co-injected fluorescently labeled histologic tracer, rhodamine-dextran-amine, was clearly visible at the injection site, confirming its precise location at the higher resolution afforded by light microscopy.

#### Pair-wise grouped voxel-wise analysis of Mn^2+^ tracings reveals functional connectivity differences between NET KO and wildtype littermates

**Figure 6:**
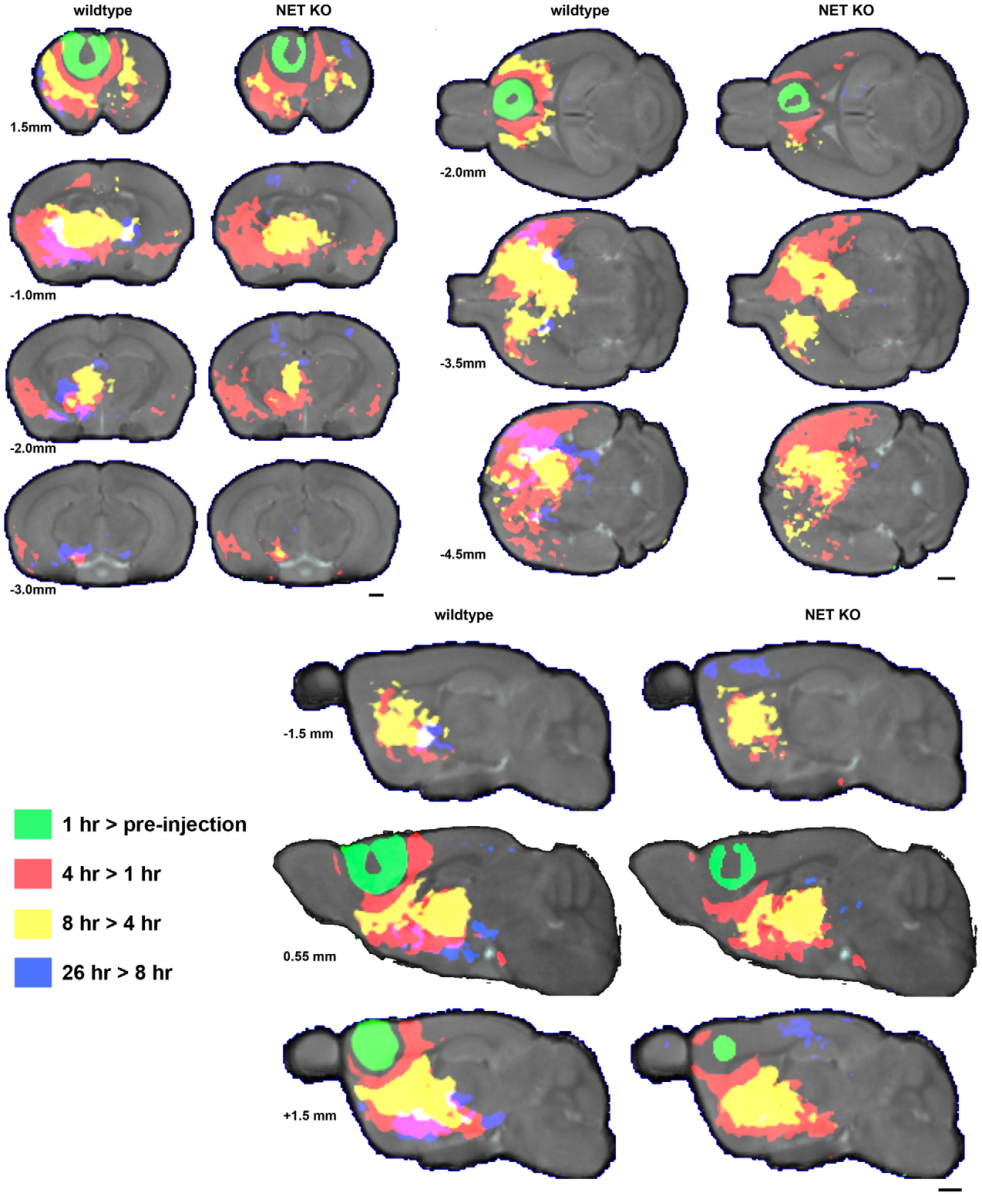
Statistical parametric maps show the progression of Mn^2+^ accumulation over time. Representative sections through the statistical parametric maps show the progression of Mn^2+^ accumulation over time. Gray background is pre-injection MDT, while the green (1 hr > pre-injection), red (4 hr >1 hr), yellow (8 hr >4 hr) and blue (26 hr >8 hr) overlays indicate areas with increased intensity (FDR corrected at p<0.001) compared to the preceding time point. Representative axial (A), sagittal (B), and transverse (C) sections are displayed. Videos in Supplemental Information show the complete image data sets in each orientation for each genotype and in 3D (S3, S4, S5). Slice locations are indicated relative to Bregma for axial, relative to midline for sagittal, and relative to the brain surface for transverse sections are indicated to the left in each panel. Specific anatomical regions can be identified using Hoff et al. [109], the Allen Brain Atlas [110] (http://www.brain-map.org/welcome.do), or The Mouse Brain Library (http://www.mbl.org/atlas/atlas.php). Scale bars  = 1 mm.

To follow the movement of Mn^2+^-induced hyperintensities in the MRI data sets, we performed image alignment and statistical parametric mapping to compare how NET KO and WT littermates differed in the time dependence of Mn^2+^ accumulation at locations distant from the injection site. This analysis reveals the progressive evolution of Mn^2+^ accumulation through multisynaptic circuitry emanating from the PFC that is detected as Mn^+2^-induced signal intensities change from one time point to the next. Three-dimensional maps can be color coded to demonstrate evolution over time (see Videos S1, S2, S3, S4, S5). Two-dimensional sections through the compiled maps superimposed on a gray-scale anatomical image showed statistically significant (FDR corrected at p<0.001) temporal increases in Mn^2+^ induced hyperintensities that differ between genotypes (Figure 6). Colored areas show progression from one time point to the next: green, 1 hr > pre-injection; red 4 hr >1 hr; yellow, 8 hr >4 hr; and blue, 26 hr >8 hr. Videos that show the entire 3D data sets are available as supplementary information (See videos S3, S4, S5).

In the SPM images that compare 1 hr post-injection images with pre-injection images, statistically significant voxels define the injection site (Figure 6, green). Comparison of 4 hr with 1 hr images showed minimal extension anteriorly from the injection site in either genotype (Figure 6, red). At 4 hr increases were seen in the cortex (S2, GI, AIP, and Pir) ipsilateral to the injection site between 1.4 mm and −1.5 mm from bregma in wildype mice and to some lesser extent in the KO mice (Figure 6, red). These increases narrowed to encompass only the piriform cortex (Pir) at −2.8 mm from bregma. Hyperintensity was also seen ipsilaterally in most of the striatum as well as portions of the bed nucleus of the stria terminalis (BST), ventral pallidum (VP), thalamus, and the anterior half of the substantia nigra (SNr). The wildtype mice also exhibited hyperintensity increases in the contralateral nucleus accumbens (NAc) and ventral striatum. The NET KO mice displayed similar increases from 1 hr to 4 hr; although there were smaller contributions in the contralateral NAc and striatum, but larger contributions in the SNr.

Progressing to 8 hr from 4 hr (Figure 6, yellow), signal intensity increased in the wildtype mice ipsilateral to the injection site in the anterior third of the striatum, medial thalamus posterior to −2.5 mm bregma, and a small fraction of the SNr. Contralateral to the injection site there were increases in the motor cortex near bregma and small portions of the medial thalamus near the midline posterior to −2 mm from bregma. Again, the NET KO mice showed a similar pattern from 4 hr to 8 hr, but with no increases in the cortex and some increases in the anterior SNr.

There were genotype dependent differences at the final time point, 25 hr post injection (Figure 6, blue). In wildtype mice hyperintensity increases ipsilateral to the injection site were observed in the ventral portions of the striatum, the ventromedial thalamus, the posterior third of the SNr, and the median raphe nucleus. Contralateral to the injection site, small increases were seen in the medial striatum and thalamic reticular nucleus (RT), but nothing posterior to 1 mm from bregma. For the NET KO mice at 25 hr, the intensity increases were less extensive than for wildtype littermates, with small areas in the ipsilateral somatosensory cortex at bregma, along the midline in the paraventricular nucleus (PVA) and in the ipsilateral posterior SNr showing increased intensity. Table 1 summarizes the Mn^2+^ induced hyperintensities that originated at the injection site and progressed to distal parts of the brain with time and enable visualization of the slower progression of Mn^2+^ towards posterior structures in the knockout animals.

**Table 1:** Comparison of anatomical structures highlighted by Mn^2+^ over time in NET KO and wildtype littermate mice; and at one day post Mn^2+^ injection in DAT and SERT KO mice and their respective wildtype controls.

Anatomical feature	1hr	4hr	8hr	25hr	DAT 26hr	SERT 25hr
PFC: prefrontal cortex	B_i_					
Cortex (S, M, AIP, Cg)		B_i_	WT_ic_	KO_c_	B_i_	
STR: Striatum		B_i_	B_ic_	WT_i_	B_ic_	WT_i_
Pir: Piriform cortex		B_ic_		WT_i_		
VP: ventral pallidum		B_ic_	KO_i_	WT_i_	B_ic_	
ACB: nucleus accumbens		B_ic_			B_ic_	WT_ic_
GP: globus pallidus		B_i_	B_ic_	WT_ic_	B_ic_	KO_i_ & WT_ic_
AMG: amygdala		B_ic_		WT_i_		
Septal nuclei			WT_i_			
TH: thalamus		B_ic_	B_ic_	WT_i_	B_ic_	
HY: hypothalamus LH					WT_ic_	KO_i_
SI: substantia innominata		WT_i_				WT_ic_
SNr: substantia nigra		B_i_	B_i_	B_i_	B_i_	KO_ic_
MG: medial geniculate nu					WT_i_	
Ml: medial lemniscus		WT_i_				
VTA: ventral tegmental area						KO_i_
RN: red nucleus						KO_i_
PRN: pontine reticular nucleus						KO_i_

Manganese accumulation over time in NET KO mice compared to wildtype littermates follows a similar general time-course as seen in the SERT and DAT KO strains. Wildtype littermates exhibit more widespread Mn^2+^ induced hyperintensities than the NET KO mice at all times post-injection in PFC. At the 24 hour time point, the pattern of manganese accumulation is significantly restricted in the NET KO compared to wildtype mice. I subscript: ipsilateral, C subscript: contralateral, WT: wildtype only, KO: knockout only, B: both wildtype & knockout.

Differences are considered significant at p<0.001 (FDR corrected) level.

## Discussion

In this study we investigated how mice with lifelong genetic deletion of the norepinephrine transporter differ from their wildtype littermates in aspects of metabolite levels, brain morphology and neuronal circuitry associated with reward pathways. We employed a panel of magnetic resonance methods with voxelwise analyses, as well as histological confirmation. This work extends earlier examinations of serotonin and dopamine transporter knockout mice [40], [41]. Interactions between the dopaminergic, serotonergic and noradrenergic systems are well known [14], [20], [22], [38]. Comparison of differences detected by MEMRI in the reward circuitry of mouse strains with deletion of each of the three monoamine transporters emphasizes the synergistic complexity of these overlapping integrated systems.

We used MRS to measure metabolite levels averaged over an 8 μl volume centered in the striatum. Only N-acetyl aspartate levels differed between the NET KO and wildtype mice. Striatal NAA was significantly, 36%, lower in NET KO than WT littermates. Alterations in fiber bundles at the striatal interface with the global pallidus are seen by histologic analysis, which may account for this decreased NAA signal as NAA is found predominantly in axons and nerve processes [70]. While NAA's cellular function and consequences of any change in concentrations remain largely un-elucidated [71], it is abundant, broadly distributed, displays a range of concentrations between different neuronal types [72] and serves as a surrogate marker for neuronal loss and/or neuronal viability [73]. Reduced brain NAA is reported in a wide range of neurological disorders, including Alzheimer's disease, post-traumatic stress disorder, mesial temporal lobe epilepsy, and schizophrenia [51], [74]–[76]. Thus, reduced NAA in the NET KO is consistent with reduced neuronal viability induced by lifelong elevated levels of norepinephrine and/or developmental consequences of disruption of NET [77]–[79]. In previous work, we observed that NAA levels in SERT KO and DAT KO mice are not significantly different from those of wildtype littermates [40], [41]. These results highlight the different ramifications of deletion of the different monoamine transporters.

There were no morphological alterations in the major structures involved in the reward pathways (*i.e.* PFC, CP, NAC, VP, AMG, VTA), although minor expansions and contractions throughout the brain were detected between wildtype littermates and NET KO mice. Similarly, in our other studies with SERT and DAT KO mice, TBM did not reveal differences between the knockout mice and wildtype littermates. Thus, structural changes at the resolution employed here do not explain differences in behavioral phenotype: compared to wildtype NET KO mice show enhanced behavioral responses to cocaine [80], [81], profound hypoalgesia [17], reduced MPTP toxicity [82], and less immobility in forced swim test and tail suspension test [22]. Moreover, functional circuitry differences identified via MEMRI are not necessarily reflected in morphology differences identified by TBM at the resolution employed here.

MEMRI employs the unique properties of Mn^2+^ to analyze functional connectivity and patterns of neuronal activity *in vivo* [48], [83]–[85]. Mn^2+^ preferentially enters active neurons, is transported along microtubules and transmitted across active synapses [85], [86]. Hyperintensity in MEMRI is a consequence of changes in local Mn^2+^ concentrations determined by at least 4 contributing factors: neuronal uptake at the injection site, packaging and anterograde axonal transport, synaptic release and re-uptake, and intracellular accumulation, likely in endoplasmic reticulum [46], [87], [88]. NET absence is unlikely to affect Mn^2+^ uptake. The effective delineation of circuitry in the NET KO group confirms satisfactory uptake of Mn^2+^. Furthermore, by comparing time points within each group, we ensure that any putative differences in uptake between genotypes would not contribute to our conclusions.

In the present work, stereotaxic injection of nanoliter amounts of Mn^2+^ into the mouse prefrontal cortex (PFC) in NET KO mice and their WT littermates revealed differences in the pattern of functional connectivity from the PFC to distal brain regions as deduced from time dependent manganese induced MRI hyperintensities far from the injection site. The lack of any remarkable pathologic changes around the injection site in histopathologic examination demonstrates that stereotaxic injection of Mn^2+^ with fluorescent tracers can be minimally invasive despite the small displacement occasioned by the co-injected oil droplet. Histology also confirmed that the injection site was positioned in the desired forebrain location. As in previous work, there was little histologic evidence of tissue damage [87].

The statistical parametric maps (SPMs) shown in Figure 6 reveal the time-course and spatial extent of the active neuronal circuitry traced out by Mn^2+^ induced hyperintensities in the MR images of wildtype and NET KO mice. Table 1 indicates which anatomic structures display Mn^2+^ induced hyperintensities as a function of time after Mn^2+^ injection into the PFC. MEMRI measures of active neuronal circuitry emanating from the prefrontal cortex follow the expected prefrontal ventrostriatal pathway that is influenced by mesolimbic and mesocortical dopaminergic projections [43], [89]–[91].

**Figure 7:**
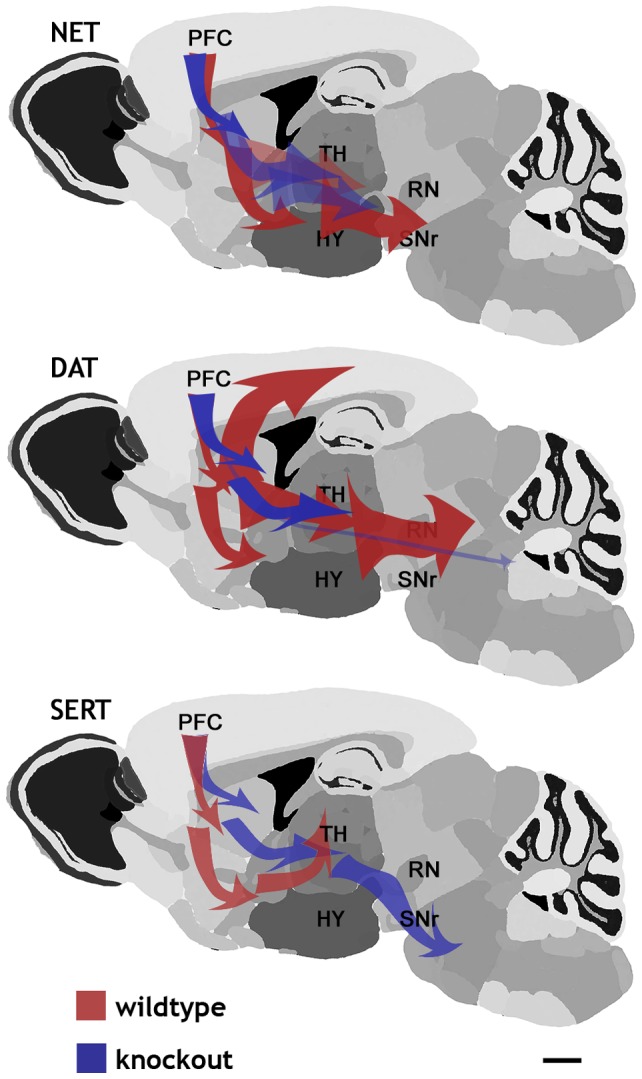
Schematic representation shows PFC associated circuits are somewhat more robust in WT compared to NET KO mice. Major circuits delineated by MEMRI are shown for NET, DAT, and SERT KO mice compared with wildtype littermates. Red (WT) and blue (DAT KO) arrows in the schematic diagram indicate Mn^2+^ transport and accumulation in the different genotypes. Detailed anatomy and timing of Mn^2+^ induced image changes are shown in Table 1.

Similarities and differences between the NET KO reported here and the other two monoamine transporter knockout strains, DAT KO and SERT KO, are displayed in the simplified schematic diagrams summarizing the SPM analyses (Figure 7). These results reveal different time courses of Mn^2+^ tracing in NET, DAT and SERT KO mice (blue tracings in Figure 7). The SERT KO MEMRI tracing extends more posteriorly than traces from their wildtype littermates. Both the DAT KO and NET KO tracings are truncated compared to those in their wildtype littermates, extending only as posterior as the SNr. The prefrontal cortical and striatal neurons normally project to multiple brain regions, including posterior regions in the midbrain and pons.

There is a growing body of literature indicating the subtleties, complexities and interrelatedness of the consequences associated with NET, DAT, and SERT deletions. NET KO can up-regulate DAT and SERT in several brain regions [34]. SERT and DAT can accumulate and release NE as a result of heterologous “false transmitter” uptake of the transmitter [38], [92], [93], although “occult” reuptake is unlikely to account for many effects observed in these mice (see discussion in Moriya et al., In Press [94]). There is considerable evidence for adaptations in the other monoamine systems when one transporter is deleted (for review see Sora et al., 2010) [95], which is perhaps best exemplified by the unusual rewarding effects of selective NET and SERT blockers in DAT KO mice [81].

The differences in the MEMRI tracing in posterior brain regions may indicate that the balance of activity between anterior and posterior projections from the PFC is altered in these monoamine knockout mice. This change in active connectivity may underlie the characteristic behavioral consequences of each of these knockouts, such as the anxiety-like phenotype of the SERT KO [96], as contrasted with antidepressant-like phenotype of the NET and DAT KO mice [22], [97] (see review by Haenisch and Bonisch [93]). Thus, the mainly frontal MEMRI tracing in DAT and NET KO mice is likely to be indicative of more robust activity and/or connectivity in the frontal portion of the reward circuit of the DAT and NET KO mice compared to wildtype or the SERT KO mice. In the context of understanding the genetic basis of mental illness, Robbins and Arnsten [98] review monoaminergic influences on executive functions of the PFC in rodents, nonhuman primates and humans. They note that much subtler genetic alterations in norepinephrine and dopamine signaling may have significant influences on susceptibility for major mental illnesses.

## Conclusions

In making comparisons across the three monoaminergic systems (DAT, SERT, NET), two caveats must be remembered: 1) there is significant cross-talk among the systems (substrates often modulate more than one transporter system) and 2) the transporter knockout mice have experienced lifelong elimination of the transporter so that both adult and developmental effects are likely. Although this is often a criticism of constitutive gene knockout approaches, it is likely that many gene effects in humans have a similar basis – that is, they are present consistently throughout development and do not suddenly appear in adulthood.

TBM analysis indicates little structural change across the genotypes examined. Only the NET KO genotype expresses a metabolite pattern different from wildtype mice with decreased NAA in the striatum, which is also reflected in the subtle dysmorphism observed histologically. Lack of aberrant behavior post stereotaxic injections and normal histology indicate that MEMRI using focal injection of Mn^2+^ in PFC is a benign methodology.

Longitudinal recording of Mn^2+^ tracings from the PFC vary in extent and intensity across the monoamine knockouts. NET and DAT KO mice are most similar with Mn^2+^ tracings from PFC restricted to the anterior portions of the reward circuit, whereas, the SERT KO mouse is the ‘outlier’ with extensive MEMRI tracing as far posterior as the PAG. These animals are important model systems in a number of affective disorders (*e.g.* addiction, pain, depression, anxiety, addiction) with complex behavioral and molecular phenotypes. MRI provides mesoscopic level information about structure and neuronal circuitry in the DAT, SERT, and NET KO brains that can be used to aid in testing mechanistic interpretations of monoaminergic system dysfunctions. Detecting similarity and differences among the monoamine transporter knockout strains with respect to functional circuitry is a promising methodology to compile a comprehensive phenotypic/genotypic understanding of reward pathways, drug use and addiction, as well as mood disorders involving the monoamine transmitters and mesolimbic circuitry.

## Materials and Methods

### Ethics Statement

All protocols involving animals in this work were approved by the Institutional Animal Care and Use Committee (IACUC) of the California Institute of Technology.

### Animals

Mice null for the norepinephrine transporter have been described previously [80], [81]. These animals have a mixed C57BL/6J –129Sv genetic background. The effects of genetic background have been controlled in these studies by using wildtype littermates from heterozygote x heterozygote crossings. While it is possible that small genomic regions around the NET locus are more likely to derive from the 129 mouse strain due to repeated selection of the knockout NET allele, examination of the genes adjacent to NET (a matrix metallopeptidase, a calpain subunit and a lysophosphatidylcholinc acyltransferase centromeric to NET and three carboxylesterase genes telomeric) provides no strong candidates that display significant influences on brain structures and connectivities. We used 11 NET KO (6 males and 5 females) and 12 wildtype littermates (6 males and 6 females). Animals were between 6–7 months of age at the time of experiments (average age of 25 weeks) and genotypes were confirmed by PCR according to standard procedures (for details see Perona et al., (2008)) [22].

### Stereotaxic Injections

The stereotaxic injection procedure was similar to that employed previously [41] and in approximately the same location in the prefrontal cortex. As Koob & Moal [99] emphasize, the prefrontal cortex (PFC) is really a system composed of the orbitofrontal, medial prefrontal, and prelimbic/cingulate cortices. We chose our injection site location to minimize spill over into non PFC structures and to be consistent with earlier work in the DAT KO and SERT KO mice [40], [41].

Briefly, 3-5 nl of 600 mM Mn^2+^, prepared with 0.5 mg/ml rhodamine dextran-amine (RDA; 3k; Molecular Probes/Invitrogen, OR, USA), was injected unilaterally into the right prefrontal cortex (coordinates *x*  = −0.8 mm (midline), *y* = 1.9 mm (bregma), *z* = −1.8 mm (down) [100]) over 5 minutes using a quartz micropipette guided by computer-assisted stereotaxic injector (myNeuroLab.com, IL, USA). Following micropipette removal, animals were transferred to the scanner and maintained under anesthesia with 1–1.5% isoflurane in medical grade air. Inspection of MR images obtained 1 hour after injection enabled identification of a hypointense region indicative of the injection site. The coordinates of this region were identified in all animals and translated to the bregma coordinate system through application of the transformation required to align the coordinates of the anterior commissure in the 1 hour MR images and the high resolution template image used throughout the study.

### Magnetic Resonance Imaging

All animals were imaged prior to administration of Mn^2+^ to acquire a pre-injection image and received 0.3 ml of saline subcutaneously before each imaging session at 0:47±0:14, 4:21±0:17, 8:10±0:13, 24:43±1:04 hours post injection; no differences in scan time were observed between the groups. The midpoint of each 46 minute scan was identified as the “scan time” and will be referred to as occurring 1, 4, 8 and 25 hours after Mn^2+^ injection.

An 11.7 T 89 mm vertical bore Bruker BioSpin Avance DRX500 scanner (Bruker BioSpin Inc., Billerica, MA) equipped with a Micro 2.5 gradient system was used to acquire all mouse brain images with a 35 mm linear birdcage radio frequency (RF) coil. During imaging the animals head was secured in a Teflon stereotaxic unit within the RF coil to minimize movement and to aid in reproducible placement. Temperature and respiration were continuously monitored during data acquisition with temperature maintained at 37°C and respiration maintained at a rate of 100–120 per minute. Similar to previous MEMRI studies [40], [41], [101], we employed a 3D RARE imaging sequence [102] with RARE factor of 4, 4 averages, TR/TE_eff_  = 250 ms/12 ms; matrix size of 160×128×88; FOV 16 mm ×12.8 mm ×8.8 mm; yielding 100 µm isotropic voxels with a 46 minute scan time.

### Fixation for Histology and Diffusion Tensor Imaging

Mice were sacrificed 9–10 days after the final MR imaging session. Each mouse was anaesthetized and perfused with warm heparinized phosphate buffered saline (PBS; 30 ml) followed by 30 ml of room temperature 4% paraformaldehyde (PFA) in PBS. Following decapitation the head was placed in 4% PFA in PBS overnight at 4°C. Samples intended for *ex vivo* MRI were kept within the skull, had all skin and cartilaginous tissue removed and were maintained at 4°C in 0.01% sodium azide in PBS before further processing prior to scanning.

### Diffusion-Weighted MRI

Two intact heads prepared as described above were secured in a Teflon® holder, submerged in perfluoropolyether (Fomblin®, Solvay Solexis, Inc., Thorofare, NJ) and imaged at 4°C. Diffusion weighted images (DWI) were acquired as previously described [40], [41] using a conventional pulsed-gradient spin echo (PGSE) sequence (TR/TE  = 300 ms/11.9 ms, 256×150×120 matrix, 25.6 mm ×15 mm ×12 mm FOV, 100 µm isotropic voxel size, 1 average, δ = 3 ms, Δ = 5.2 ms, G_d_  = 1125 mT/m, nominal b-factor  = 3370 s/mm^2^). An optimized six point icosahedral encoding scheme [103] was used for diffusion weighted acquisitions with a single un-weighted reference image for a total imaging time of 14.5 hours.

### Histology

Histological examination was performed on 6 brains, 3 NET KO and 3 wildtype littermates. Prior to processing, each brain was removed from the skull, post-fixed for 1–3 days and sent to Neuroscience Associates (NSA, TN, USA) for gelatin embedding and frozen sectioning as described [40]. Brains were embedded in a single gelatin block and sectioned in register at 30 µm thickness. Every 12^th^ section was stained with a Solochrome technique, developed for our studies with NSA, and an adjacent section mounted in DAPI-containing anti-quench and mounted for fluorescence microscopy of the co-injected rhodamine-dextran. Microscopy of whole brain sections was performed using a Zeiss V8 stereoscope equipped with an Axiocam running AxioVision 4.8, bright and dark-field illumination and three fluorescent filter cubes for DAPI, TRITC, and FITC. High magnification assessment of rhodamine-dextran-amine and DAPI at the injection site were obtained on a Zeiss Axioscope Z1 with an MRM and HRC Axiocam running AxioVision 4.6 software. Microscopy images were prepared for publication figures using Adobe Photoshop.

### Image Alignment, Processing and Statistical Parametric Mapping

Image alignment and statistical parametric mapping were performed as in previous work with dopamine transporter knockout mice [41]. Briefly, images were stripped of non-brain material [104], corrected for B_1_ field inhomogeneities and each scaled to the mode of its intensity histogram [68], [105], [106]. A minimum deformation template (MDT) was produced from the pre-injection images as described [40], [107]. The pre-injection images were then warped into this MDT as described [40] and the post-injection images were linearly aligned (12 parameter model) to the pre-injection images using Alignlinear, followed by application of the polynomial warp field used to transform that sample's pre-injection image into the MDT. Final images were blurred with a 0.3 mm Gaussian kernel and a paired Student's t-test with false discovery rate (FDR) correction was used to determine which voxels increased in intensity when comparing one time point to the next. Statistical significance was considered reached at a False Discovery Rate (FDR)-corrected p<0.001. Parametric maps of voxels with statistically significant changes in intensity were created to display the results and to correlate increases with underlying anatomy [106]. All processing after skull stripping was performed using the LONI pipeline environment [108]. Anatomy was determined with reference to Hoff et al. [109] and the Allen Brain Atlas [110] (http://www.brain-map.org/welcome.do).

### In vivo Magnetic Resonance Spectroscopy

I*n vivo* mouse brain magnetic resonance spectroscopy (MRS) employed Point Resolved Spectroscopy (PRESS) [111] with a short echo time TR/TE  = 2.3 s/7.27 ms, with a spectral width of 7 KHz, 4000 data points in each free induction decay signal (FID), and 128 averages. The sequence was preceded by a VAPOR water suppression module [112] interleaved with outer volume saturation. Optimized second order shimming was done with the FastMap routine [113] in a 5 mm cube centered in the striatum. The PRESS spectra were then recorded inside a (2 mm)^3^ volume (8 μl) in the striatum at the center of the volume used for shimming.

For each mouse brain (n = 11 NET KOs and n = 12 wildtype) the spectrum of the relative amount of metabolites inside the experimental PRESS volume was quantified using the QUEST (quantitation based on quantum estimation) module [114] available inside the Java Magnetic Resonance User Interface (JMRUI) package [115]. Spin parameters (number of spins, chemical shifts, J-couplings) were obtained from Govindaraju [116]. Metabolite amounts obtained by the QUEST were normalized to creatine plus phosphocreatine.

### Tensor-based morphometry

Six icosahedrally directed diffusion weighted images were averaged to generate a high CNR isotropic diffusion weighted image (iDWI). A deformation field analysis implemented by Dinov, Thompson and coworkers [117], [118] was used to analyze morphological differences between 8 NET KO images and 12 wildtype litermate brain images; processing was implemented in the LONI Pipeline Processing Environment. All scans were mapped into the wildtype iDWI minimum deformation template (MDT) and the Jacobian determinant of the displacement field (det|J^T^J|, with J the deformation Jacobian) for each scan determined. An FDR corrected independent samples *t*-test assessing the difference between the absolute value of the determinants of the displacement fields of the wildtype and NET KO identified areas of local expansion and shrinkage [117]. Statistical significance was considered reached at an FDR-corrected p<0.005.

## Supporting Information

Figure S1 **Representative examples of brain sections stained for choline acetyl transferase (ChAT) from one of the wildtype (NET+/+) and one of the NET knockout (NET−/−) mice analyzed by MRI in this study.** Note that 9 ChAT-stained neurons appear in the lower 1/3 of the globus pallidis (GP) in the NET−/− whereas only one is apparent in the NET +/+. Scale bar  = 200 µm.
(TIF)

Video S1 **Sections through statistical parametric maps show local changes in the determinant of the deformation tensor: det|J|, where J is the deformation Jacobian.** Regions showing significant (FDR corrected p<0.005) expansion of KO relative to wildtype (det|J|<0) are shown in blue and contractions ((det|J|>0) in red with the color scale indicating extent of fractional change in local volume. Gray background is pre-injection MDT.
(MPG)

Video S2 **Axial sections.**
(MPG)

Video S3 **Sagittal sections.**
(MPG)

Video S4 **Transverse sections.**
(MPG)

Video S5 **Three dimension rendering.** Sections through the statistical parametric maps show the progression of Mn^2+^ accumulation over time. Gray background is pre-injection MDT, while the colored overlays denote areas with increased intensity (FDR corrected p<0.001) at 1 hr (green), 4 hr (red), 8 hr (yellow), and 24 hr (blue) compared to the preceding time point. Videos show consecutive sections in the axial, sagittal, and transverse directions for the NET KO and wildtype cohorts. Slice locations are shown at the bottom right in millimeters with respect to Bregma, midline, and the brain surface for axial, sagittal, and transverse sections, respectively. Scale bar  = 1 mm.
(MP4)
