# Correction: Altered Reward Circuitry in the Norepinephrine Transporter Knockout Mouse

**DOI:** 10.1371/annotation/76516adc-5541-4941-b4ff-e80d1c4d769f

**Published:** 2013-08-16

**Authors:** Joseph J. Gallagher, Xiaowei Zhang, F. Scott Hall, George R. Uhl, Elaine L. Bearer, Russell E. Jacobs

Two additional grants from the NIH to the fifth author (ELB) were incorrectly omitted from the Funding Statement. The numbers of the two grants are: NIMH R01 MH096093 and NINDS R01 NS062184. 

