# Supplementary figures and images for: Altered Reward Circuitry in the Norepinephrine Transporter Knockout Mouse
Source: PLoS One. 2013 Mar 4;8(3):e57597. doi: 10.1371/journal.pone.0057597 (PMC3587643; doi:10.1371/journal.pone.0057597)

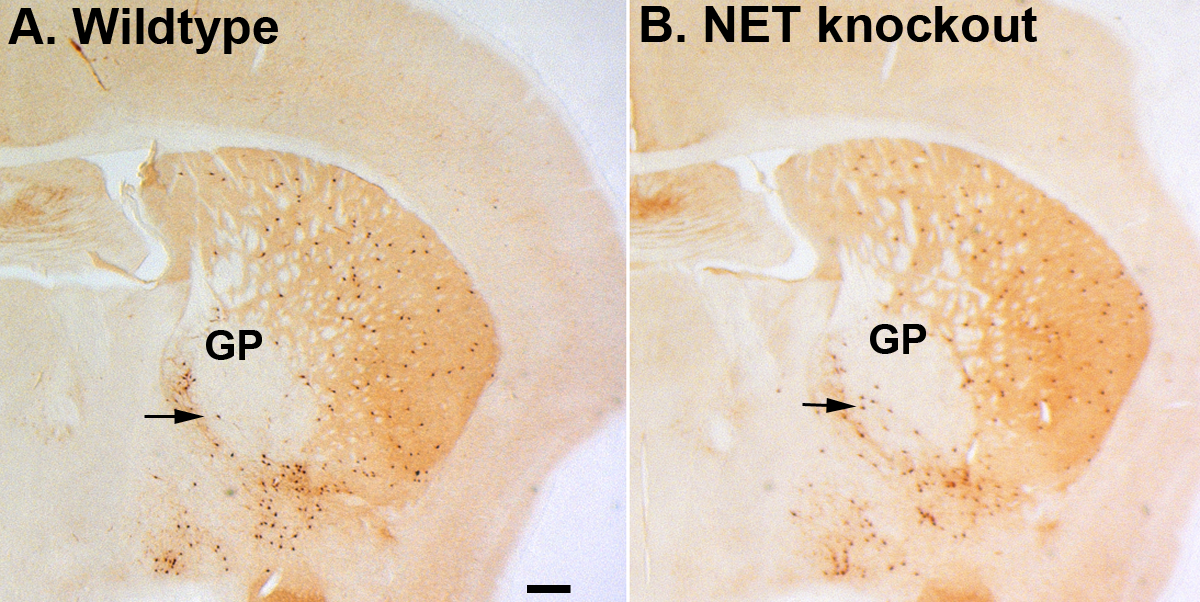

Supplement: Figure S1 — Representative examples of brain sections stained for choline acetyl transferase (ChAT) from one of the wildtype (NET+/+) and one of the NET knockout (NET−/−) mice analyzed by MRI in this study. Note that 9 ChAT-stained neurons appear in the lower 1/3 of the globus pallidis (GP) in the NET−/− whereas only one is apparent in the NET +/+. Scale bar = 200 µm. (TIF) [file pone.0057597.s001.tif]
